# Left Atrial Pressure as a Predictor of Success in Catheter Ablation of Atrial Fibrillation in a Real-Life Cohort

**DOI:** 10.3390/jcm10153208

**Published:** 2021-07-21

**Authors:** Massimiliano Manfrin, Giacomo Mugnai, Werner Rauhe, Vedran Velagic, Matthias Unterhuber

**Affiliations:** 1 Electrophysiology and Cardiac Pacing Unit, San Maurizio Regional Hospital, 39100 Bolzano, Italy; massimiliano.manfrin@sabes.it (M.M.); werner.rauhe@sabes.it (W.R.); 2 Electrophysiology and Cardiac Pacing Unit, Division of Cardiology, West Vicenza General Hospitals, 36071 Arzignano, Italy; mugnai.giacomo@gmail.com; 3 Department of Cardiovascular Diseases, University of Zagreb School of Medicine, University Hospital Centre Zagreb, 10000 Zagreb, Croatia; vvelagic@gmail.com; 4 Heart Center Leipzig, Department of Internal Medicine/Cardiology, University of Leipzig, 04289 Leipzig, Germany

**Keywords:** left atrial hypertension, atrial fibrillation, pulmonary vein isolation, catheter ablation

## Abstract

Aims: The clinical role of the left atrial (LA) hypertension in patients with atrial fibrillation (AF) and its role as predictor in those undergoing pulmonary vein (PV) isolation is still unknown. The aim of the present study was to analyze the role of LA pressure in patients with nonvalvular AF who underwent PV isolation and its implication for AF catheter ablation. Methods: Consecutive patients with drug resistant AF who underwent PV isolation at San Maurizio Regional Hospital of Bolzano (Italy) as index procedure were included in this analysis. Results: A total of 132 consecutive patients (97 males, 73%; mean age 58.0 ± 13.2 years) were included in the analysis. Eleven patients (8%) underwent radiofrequency ablation and 121 (92%) cryoballoon ablation. Higher LA pressures were found in 54 patients (40.9%). At a mean follow up of 14.3 ± 8.2 months (median 12 months), the success rate without antiarrhythmic therapy was 65.9% (87/132; considering the blanking period). Female gender and continuous mean LA pressure were significantly associated with AF recurrence and remained significant on multivariable Cox analysis (respectively, HR 1.845, 1.00–3.40, *p* = 0.05 and HR 1.066, 1.002–1.134, *p* = 0.04). We identified a LA mean pressure of >15 mmHg as ideal cutoff and constructed a model to predict AF recurrence which fitted with a concordance index (C-index) of 0.65 (95% CI 0.56–0.75), logrank score *p* = 0.003.

## 1. Introduction

Atrial fibrillation (AF) is the most common rhythm disturbance in western society. Pulmonary vein (PV) isolation remains the cornerstone for the treatment of symptomatic drug-refractory paroxysmal AF [1]. The pathophysiology of AF is multifactorial and includes aging, hemodynamic stress, biochemical factors, and associated structural heart disease [2]. The perpetuation of AF results in progressive electrical and structural remodeling of the left atrium (LA) [3,4]. The current estimation of LA disease is commonly based on LA volume, AF duration, presence of comorbidities, associated heart diseases, cardiac imaging, and voltage mapping. A specific direct assessment of the LA pressure might provide an additional, more complex, and complete information on the LA status [5].

The increased atrial wall stress, a consequence of left atrial hypertension and chamber dilation, results in apoptosis and interstitial fibrosis. Extensive atrial remodeling is associated with more advanced forms of AF, such as the development of persistent AF, resistance to rhythm control strategies, and recurrences after catheter ablation [6,7]. 

In this study we investigated the role of LA pressure in a sample of patients having undergone catheter ablation of AF in order to analyze the prevalence of LA hypertension in this population and to understand its role as a possible predictor of AF recurrence following the PV isolation.

As a matter of fact, the recognition of patients with higher LA pressures might help to identify a specific subgroup of patients who have a more diseased left atrium potentially leading to a worse outcome following AF ablation, and therefore who might hypothetically benefit from a different strategy of the ablation. 

## 2. Methods

Consecutive patients with drug resistant paroxysmal or either persistent or long persistent AF, who underwent PV isolation from 2017 to 2019 at the Electrophysiology and Cardiac Pacing Unit of San Maurizio Regional Hospital of Bolzano (Italy), were retrospectively included in this analysis as index procedure. All patients signed informed consent for the procedure. The protocol was carried out in accordance with the ethical principles for medical research involving human subjects established by the Declaration of Helsinki protecting the privacy of all participants, as well as the confidentiality of their personal information. The exclusion criteria included the presence of intracavitary thrombus, uncontrolled heart failure, cardiomyopathy, congenital heart disease, or valvular heart disease.

Prior to the procedure, all patients underwent a 2D transthoracic echocardiogram (TTE) to assess left ventricular ejection fraction and to rule out any structural and/or valvular disease. 

Catheter ablation was performed under conscious sedation using both 3-dimensional navigation (Ensite Precision^TM^, NavX, Abbott, St. Paul, MN, USA) with point-by-point radiofrequency ablation (3.5-mm FlexAbility^TM^ ablation catheter, Abbott, St. Paul, MN, USA) and cryoballoon ablation technology (Arctic Front Advance, Cryocath, Medtronic, MN, USA). All patients underwent circumferential pulmonary vein isolation. The LA pressure did not determine the ablation strategy. The ablation procedures have been described in detail elsewhere [8,9].

Left atrial pressure was measured at the end of the procedure, with patient in sinus rhythm, through the transeptal sheath placed in the LA cavity [10]. The pressure transducer was zeroed at the midthoracic level. Left atrial mean pressure was calculated over several breath cycles (30 s of measurement). All maximum, minimum, and mean values of LA pressure were recorded. Following the results provided by Park et al. [2], a maximum LA pressure ≥19 mmHg was taken as a cut-off for high LA pressure. As there are no prior published outcome-relevant mean LA pressure cut-offs, we calculated a ROC to identify the ideal cut-off value using the Youden index.

Patients were discharged the day after ablation if clinical status was stable. After the intervention, patients were continuously monitored with ECG telemetry for at least 18 h. Before hospital discharge, all patients underwent transthoracic echocardiography in order to exclude pericardial effusion. Oral anticoagulation was restarted the evening of ablation and continued for at least 3 months. In all patients, antiarrhythmic drugs were discontinued at the moment of discharge after ablation. Decision to restart AADs after the blanking period was usually made in case of a first episode of recurrence of AF.

After discharge from the hospital, patients were scheduled for follow-up visits at 3, 6, and 12 months. Twenty-four h Holter recordings were obtained at each follow-up visit. All reports of Holter monitoring or ECG recordings performed in referring centers were sent to the Electrophysiology and Cardiac Pacing Unit of San Maurizio Regional Hospital of Bolzano for diagnosis confirmation during the follow-up. Furthermore, patients were also contacted by telephone during follow up. Recurrent atrial arrhythmia (AF and atrial tachycardia) was defined as any documented symptomatic or asymptomatic sustained episode of AF or atrial tachycardia >30 s duration. A blanking period of 3 months was applied. Repeat ablation at any time was classified as recurrence. No patient was lost at follow up and all underwent the required outpatient visits and monitoring.

Continuous variables are expressed as mean ± SD or median and range, as appropriate. Categorical variables are expressed as absolute and relative frequencies. Comparisons of continuous variables were done with a Student’s t-test or the Mann-Whitney U-test, as appropriate. The Chi-square test or the Fisher’s exact test was used to compare categorical variables, as appropriate. Predictors of arrhythmia recurrence were performed using Cox proportional hazards regression models. Using receiver operator characteristic calculations, an ideal cut-off for the mean LA pressure was identified. A Cox proportional hazards model was constructed to assess the predictive ability of the variables, which yielded significant results in the univariable analysis by step-by-step forward inclusion method. The model’s goodness of fit was calculated using the concordance index and logrank tests. A 2-tailed probability value of <0.05 was deemed significant. Statistical analyses were conducted using the SPSS software (SPSS v20, Chicago, IL, USA).

## 3. Results

A total of 132 consecutive patients (97 males, 73%; mean age 58.0 ± 13.2 years) with drug resistant, nonvalvular AF, having undergone index PV isolation procedure, were included in the analysis (Table 1). The majority of patients (*n* = 94, 71%) had paroxysmal AF, 38 (29%) patients had persistent AF, 8 patients (6%) had long-standing persistent AF. None of the patients had prior hospitalizations for heart failure or diuretic therapy. Eleven patients (8%) underwent radiofrequency ablation and 121 (92%) cryoballoon ablation. Acute procedural success with complete PV isolation was achieved in all the procedures. The mean LA volume was 38.6 ± 15.7 mL/m^2^; persistent AF was identified in 38 patients (29%), paroxysmal AF in 94 (71%). The mean LA pressure was 10.7 ± 4.6 mmHg.

Clinical follow up was obtained in all patients. At a mean follow up of 14.3 ± 8.2 months (median 12 months), the success rate without antiarrhythmic therapy was 65.9% (87/132) in the total study population and considering the blanking period.

High LA pressures (defined as peak of LA pressure ≥19 mmHg) were found in 54 patients (40.9%). As Table 2 shows, patients with high LA pressure were significantly older (60.8 ± 8.4 vs. 56.8 ± 9.5 mmHg; *p* = 0.01) and tended to have more persistent AF (37% vs. 23%; *p* = 0.1). No differences in LA volume, gender, or cardiovascular risk factors were found between the two groups. When summarized by mean LA pressures >15 mmHg, the patients within the higher mean LA pressures group had significantly more persistent AF.

As Table 3 shows, the AF recurrences occurred numerically more frequently among women (36% vs. 22%, *p* = 0.07) and were significantly associated with mean, maximum, and minimum values of LA pressure, which were all significantly higher in patients presenting AF recurrence (respectively, 12 ± 5.0 vs. 10.2 ± 4.2 mmHg, *p* = 0.02; 21.0 ± 9.0 vs. 18.2 ± 7.3 mmHg, *p* = 0.05; 7.5 ± 3.9 vs. 5.9 ± 3.2, *p* = 0.017). Of note, age, persistent AF, and LA volume were not significantly associated with AF recurrence.

**Figure 1:**
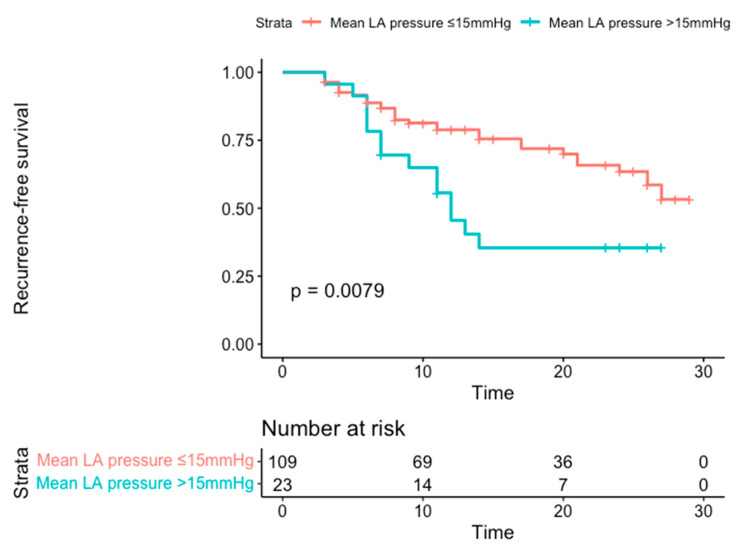
Kaplan-Meier survival curves for patients dichotomized in mean left atrial pressure >15 mmHg and ≤15 mmHg according to the receiver operating characteristics.

Table 4 shows that female gender and higher levels of mean LA pressure were significantly associated with AF recurrence on univariable analysis and remained significant predictors of AF recurrence on multivariable analysis (respectively, female: HR 1.845, 1.001–3.400, *p* = 0.05; mean LA pressure: HR 1.063, 1.001–1.129, *p* = 0.04). A mean LA pressure of >15 mmHg, as identified by the ROC Curve with an AUC of 0.63, yielded a HR in multivariable analysis of 2.43 (1.29–4.58). The cutoff value had a sensitivity of 31% and a specificity of 85%, with a positive predictive value of 58% and a negative predictive value of 70%. Of note, persistent AF and LA volume did not show any significant relationship with AF recurrence. The predictive value of the cut-off LA-mean pressure of >15 mmHg is shown in Kaplan-Meier survival curves in Figure 1.

## 4. Discussion

The main findings of the present study are: (1) higher LA pressure was found in 40.9% of patients undergoing PV isolation and was significantly associated with an older age, (2) female gender and mean LA pressure were significantly associated with AF recurrence following catheter ablation, (3) a cut-off value of mean LA pressure of >15 mmHg and female gender were associated with higher AF recurrence probability.

High LA pressures are probably the final common pathway of multiple atrial pathophysiological impairments. Increased atrial wall stress, secondary to atrial hypertension and chamber dilation, leads to apoptosis and interstitial fibrosis. Several cytokines such as angiotensin II, transforming growth factor–B, and platelet-derived growth factor are probably involved in this pathophysiological pathway [11].

Atrial fibrillation has a strict relationship with atrial structural remodeling and is often associated with fibrosis/scarring and dilatation. Substrate progression is a multifactorial and time-dependent response of cardiac myocytes to several electrical, mechanical, and metabolic “stressors” [12]. Several risk factors have a causal relationship with AF by causing structural remodeling. The effective management of risk factors such as sleep apnea, obesity, high blood pressure, hyperglycemia, and dyslipidemia has been shown to significantly prevent AF recurrence, probably by decreasing further damage and/or reversing existing abnormalities. Conversely, AF itself can lead to a significant progression of the substrate. In addition to complexion-channel remodeling that accelerates repolarization and alters conduction properties, rapid activation of atrial cardiomyocytes results in pro-fibrotic changes in fibroblast function promoting atria fibrosis [12]. Increased LA scar is also associated with increases LA stiffness resulting in a deteriorated reservoir function. Therefore, LA stiffness could be associated with LA histological changes and predicts sinus rhythm maintenance after treatment in AF patients. A systematic review and meta-analysis by Correia et al. [13] found that LA stiffness could be a predictor of AF recurrence after catheter ablation. Indeed, left atrial pressure mirrors the extent of atrial stretch, which promotes both ectopic activity and reentry. Left atrial pressure might represent an additional and important parameter of LA stiffness and remodeling, reflecting the degree of the atrial disease, and it can be easily assessed after the transeptal puncture. Therefore, the evaluation of LA pressure as a marker of stiffness might hypothetically help to learn more about the “state of health” of the LA itself and might be a predictor of catheter ablation success.

Reliable prediction of PV isolation success is crucial for both selection of eligible patients and planning of the procedural strategy of PV isolation, such as ablation of complex fractionated atrial electrograms or linear ablation, and for guiding the post-procedural follow-up decisions. Left atrial pressure is a quickly obtainable, inexpensive, and observer-independent parameter, which can be easily measured on site through transseptal sheath after PV isolation using the standard technical equipment.

Our group recently published a pilot feasibility study in a smaller sample of patients [10]. Those preliminary results showed that older age, LA volume, and LA pressure predicted AF recurrence during the blanking period but only the LA pressure was significantly associated with definitive AF recurrence following catheter ablation. Park et al. [2] investigated the role of peak LA pressure in a large cohort of patients (454) having undergone AF ablation. They found that elevated peak LA pressures were closely associated with electroanatomical remodeling of LA and were an independent predictor for clinical recurrence after catheter ablation of AF. Of note, they also found a relationship between peak LA pressure and a significant impairment of left ventricular diastolic function. Sramko et al. [5] also analyzed the role of LA pressure in patients undergoing catheter ablation of AF. They enrolled 240 patients with paroxysmal and persistent AF, preserved left ventricular ejection fraction, and found no significant valvular disease. Of note, LA pressure was measured at rest and during exercise, which involved isometric hand grip during the ablation procedure. Higher values of LA pressure, either at rest or with exercise, independently predicted AF recurrences after ablation over a mean follow-up of 16 months. In our study, we found that mean LA pressures were predictive in a continuous fashion and at rest, in contrast to the aforementioned study.

Although the majority of studies reported that patients with increased LA pressure were more likely to be female and have hypertension, diabetes and persistent AF, the present data showed no association between LA pressure and hypertension, type of AF and LA volume. However, patients with higher LA pressure were found to be older than those with normal LA pressure. Notably, both persistent AF and LA volume were not able to predict AF recurrences; on the contrary, LA hypertension expressed by a higher mean LA pressure was significantly and independently associated with AF recurrence. The missing association between persistent AF and the outcome could be due to manifold reasons. First, patients with persistent AF were a small subgroup (29%) with an AF duration of a few weeks to a few months, whereas the long-standing persistent patients were 6%. This could have led to not sufficient statistical power for confirming persistent AF as a predictor for AF recurrence in larger, specifically designed trials. Interestingly, in the present study female gender was significantly associated with AF recurrence following catheter ablation. This finding has also been described in previous studies and seems to be related to several factors [14]. First, women have a higher incidence of heart failure with preserved ejection fraction which leads to elevated filling pressures and upstream effects on LA pressure resulting in electrical and structural atrial remodeling. Second, women also have higher circulating inflammatory cytokines and expression of proinflammatory genes, including C-reactive protein. Gender-based differences in myocardial gene expression showed that many of the differentially expressed genes are involved in inflammation and cardiac remodeling. Finally, women were also found to present more advanced LA substrate with delayed enhancement on cardiac magnetic resonance imaging [14].

We recursively identified a cut-off value of a mean LA pressure of 15 mmHg, which showed a good discriminatory performance in our cohort. Interestingly, these findings are in line with previous published data [5,15] and once again confirm their validity in an unselected, all-comer, and real-life cohort for outcome prediction.

Our results, however, might have been limited by the relatively low number of patients included in the study. Nevertheless, as highlighted in previously published studies, LA pressure represents a useful, important, objective (operator-independent) indicator of LA disease. This parameter can be easily assessed with the routinary equipment during the catheter ablation procedure. It can be hypothesized that the indirect measurement of LA pressure through non-invasive techniques such as Doppler echocardiography might be extremely useful to select eligible patients for catheter ablation procedures and to guide the post-procedural follow-up strategy.

The most important limitations of this study are related to its retrospective and observational design, and consequently the lack of randomization. In addition, as all data have been collected in a single center, results should not be generalized; on the contrary, they can be of advantage by including an all-comer cohort with mixed AF types and being a real-life cohort. Another limitation is the relatively low event number, which might have limited the statistical power. In addition, LA pressure was measured after the ablation and after sinus rhythm was restored, which could have led to a further potential bias in over- or underestimating pressures possibly due to a stunned myocardium and/or fluid overload due to irrigated catheters. The study included a substantial part of paroxysmal AF patients with a smaller group of persistent and long-standing persistent AF patients. Due to this, statistical power in this subgroup could not be achieved to confirm or confute the finding of persistent AF being a predictor for AF recurrence. Finally, LA pressure was not assessed during isometric handgrip exercise; thus, some concealed LA hypertension may have been missed.

## 5. Conclusions

Higher LA pressures were found in 41% consecutive, all-comer patients of a real-life cohort with mixed paroxysmal, persistent, and long-persistent AF undergoing PV isolation and were significantly associated with an older age. Female gender and higher mean LA pressures were significantly associated with AF recurrence following catheter ablation.

## Figures and Tables

**Table 1:** Baseline characteristics of the study population.

Number of Patients	132
Age (years)	58.0 ± 13
Female gender	35 (27%)
Persistent atrial fibrillation	38 (29%)
Hypertension	53 (40%)
Dyslipidemia	12 (9%)
Diabetes mellitus	3 (2%)
Coronary artery disease	4 (3%)
Left atrial volume (mL/m^2^)	38.6 ± 15.7
CHA_2_DS_2_-VASc score	1.15 ± 0.88
Body mass index (kg/m^2^)	26.4 ± 3.5
Chronic obstructive pulmonary disease	4 (3%)
Maximum left atrial pressure (mmHg)	19.2 ± 8.0
Mean left atrial pressure (mmHg)	10.7 ± 4.6
Minimum left atrial pressure (mmHg)	6.5 ± 3.5
Mean follow up duration (months)	14.3 ± 8.2

Categorical variables are expressed as absolute and percentage (in brackets). Continuous variables are expressed as mean ± SD.

**Table 2:** Comparison of clinical characteristics between patients with high left atrial pressures (peak of left atrial pressure ≥19 mmHg and mean pressure >15 mmHg).

	LA Peak Pressure ≥19 mmHg (*n* = 54)	LA Peak Pressure <19 mmHg (*n* = 78)	*p*-Value
Age (years)	61 ± 8	57 ± 10	0.01
Male gender	43 (80)	54 (69)	0.2
Persistent AF	20 (37)	18 (23)	0.1
Hypertension	20 (37)	33 (42)	0.6
Dyslipidemia	5 (9)	7 (9)	1.0
Diabetes mellitus	2 (4)	1 (1)	0.6
LA volume (mL/m^2^)	39 ± 10	38 ± 9	0.3
	**LA Mean Pressure > 15 mmHg (*n* = 109)**	**LA Mean Pressure ≤15 mmHg (*n* = 23)**	***p*-Value**
Age (years)	58 (51–65)	64 (54–68)	0.12
Male gender	79 (72)	18 (78)	0.6
Persistent AF	27 (25)	11 (48)	0.026
Hypertension	44 (40)	9 (39)	1.0
Dyslipidemia	10 (9)	2 (9)	1.0
Diabetes mellitus	1 (1)	2 (9)	0.08
LA volume (mL/m^2^)	35 (30–47)	42 (39–45)	0.6

Categorical variables are expressed as absolute and percentage (in brackets). Continuous variables are expressed as mean ± SD or median (interquartile range) where appropriate. AF: atrial fibrillation. LA: left atrial.

**Table 3:** Comparison of clinical characteristics between patients presenting AF recurrence.

	AF Recurrence (*n* = 45)	No AF Recurrence (*n* = 87)	*p*-Value
Age (years)	59.4 ± 9.6	57.9 ± 9.1	0.4
Female gender	16 (36%)	19 (22%)	0.07
Persistent AF	14 (31%)	24 (28%)	0.4
Hypertension	16 (36%)	37 (43%)	0.5
Dyslipidemia	4 (9%)	8 (9%)	1.0
Diabetes mellitus	2 (4%)	1 (1%)	0.3
LA volume (mL/m^2^)	39.5 ± 22.0	38.5 ± 10.8	0.7
LA pressure, max (mmHg)	21.0 ± 9.0	18.2 ± 7.3	0.05
LA pressure, min (mmHg)	7.5 ± 3.9	5.9 ± 3.2	0.017
Mean LA pressure (mmHg)	12.0 ± 5.0	10.2 ± 4.2	0.02

Categorical variables are expressed as absolute and percentage (in brackets). Continuous variables are expressed as mean ± SD. AF: atrial fibrillation. LA: left atrial. Max: maximum. Min: minimum.

**Table 4:** Univariate and multivariate Cox regression analysis for AF recurrence.

	Univariable Analysis			Multivariable Analysis		
	HR	95%CI	*p* Value	HR	95%CI	*p* Value	
Age (years)	1.021	0.99–1.06	0.2				
Hypertension	1.450	0.79–2.68	0.2				
Dyslipidemia	0.720	0.26–2.03	0.5				
Female gender	1.830	0.99–3.37	0.05	1.845	1.00–3.40	0.05	
Persistent AF	1.23	0.65–2.34	0.5				
LA volume (mL/m^2^)	0.97	0.89–1.07	0.6				
Mean LA pressure (mmHg)	1.063	1.001–1.129	0.04	1.066	1.002–1.134	0.04 *	
LAmean >15 mmHg	2.31	1.228–4.349	0.009	2.43	1.29–4.58	0.006	

AF: atrial fibrillation. HR: hazard ratio. CI: confidence intervals. LA: left atrial. * The continuous LA mean variable is shown for presentation purposes, however in the multivariable model it was not included due to multicollinearity with the binary LA mean >15 mmHg parameter.

## Data Availability

The data that support the findings of this study are available from the corresponding author, M.U., upon reasonable request.
